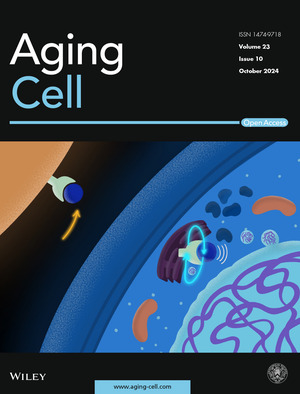# Featured Cover

**DOI:** 10.1111/acel.14378

**Published:** 2024-10-09

**Authors:** Florencia Herbstein, Melanie Sapochnik, Alejandra Attorresi, Cora Pollak, Sergio Senin, David Gonilski‐Pacin, Nicolas Ciancio del Giudice, Manuel Fiz, Belén Elguero, Mariana Fuertes, Lara Müller, Marily Theodoropoulou, Lucas B. Pontel, Eduardo Arzt

## Abstract

Cover legend: The cover image is based on the Article *The SASP factor IL‐6 sustains cell‐autonomous senescent cells via a cGAS‐STING‐NFκB intracrine senescent noncanonical pathway* by Florencia Herbstein et al., https://doi.org/10.1111/acel.14258